# Supplementary material for: Comparative Transcriptome and Interaction Protein Analysis Reveals the Mechanism of IbMPK3-Overexpressing Transgenic Sweet Potato Response to Low-Temperature Stress
Source: Genes (Basel). 2022 Jul 14;13(7):1247. doi: 10.3390/genes13071247 (PMC9317282; doi:10.3390/genes13071247)
Supplement: Supplementary file 1 [file genes-13-01247-s001.zip › genes-1758757-supplementary/(M2) genes-1758757-supplementary/Supplement documents (Figure S1-S3).pdf]

Supplement Figures

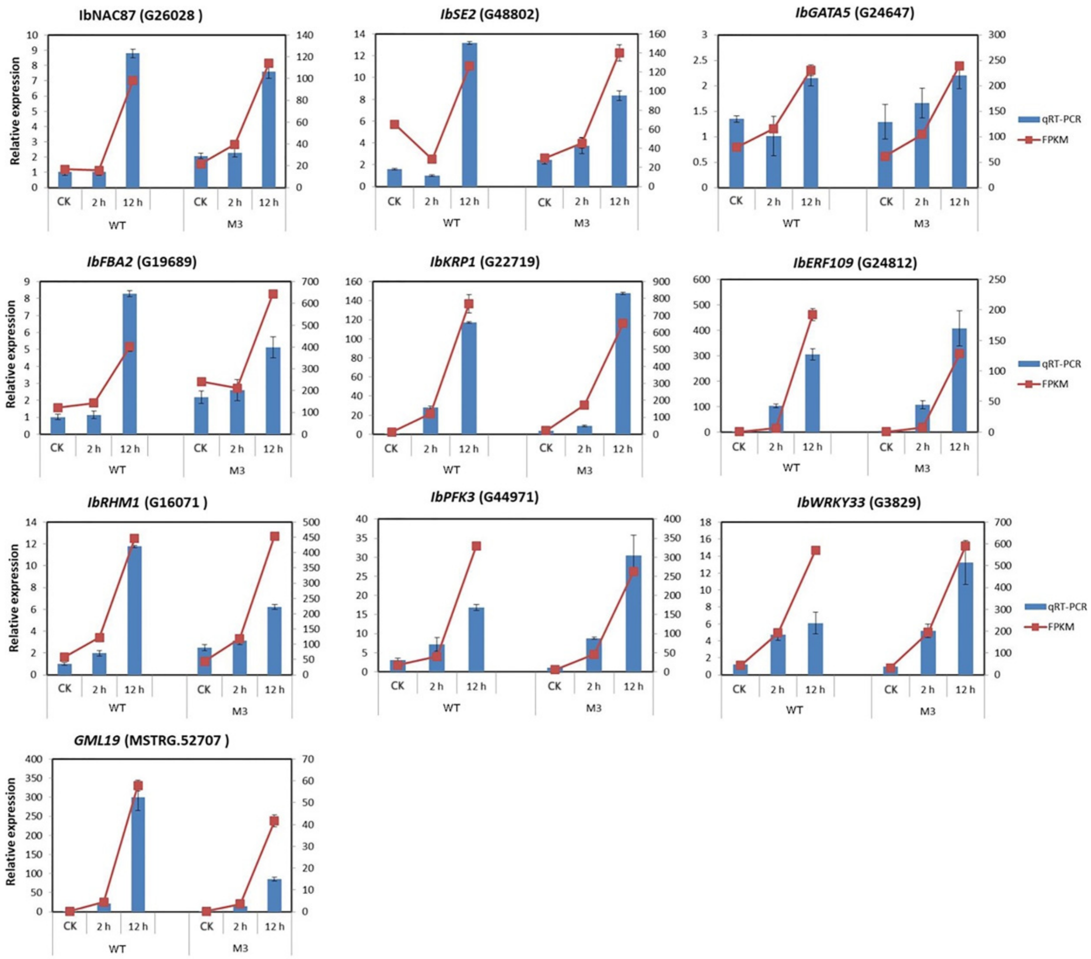

Figure S1 Confirmation of transcript levels of 10 randomly selected genes by qRT-PCR analysis. The columns represent relative expression obtained by qRT-PCR, and solid lines represent relative expression obtained by RNA-seq. All qRT-PCR reactions were performed using three biological replicates. Gene-specific primers used for qRT-PCR are listed in Table S 18.

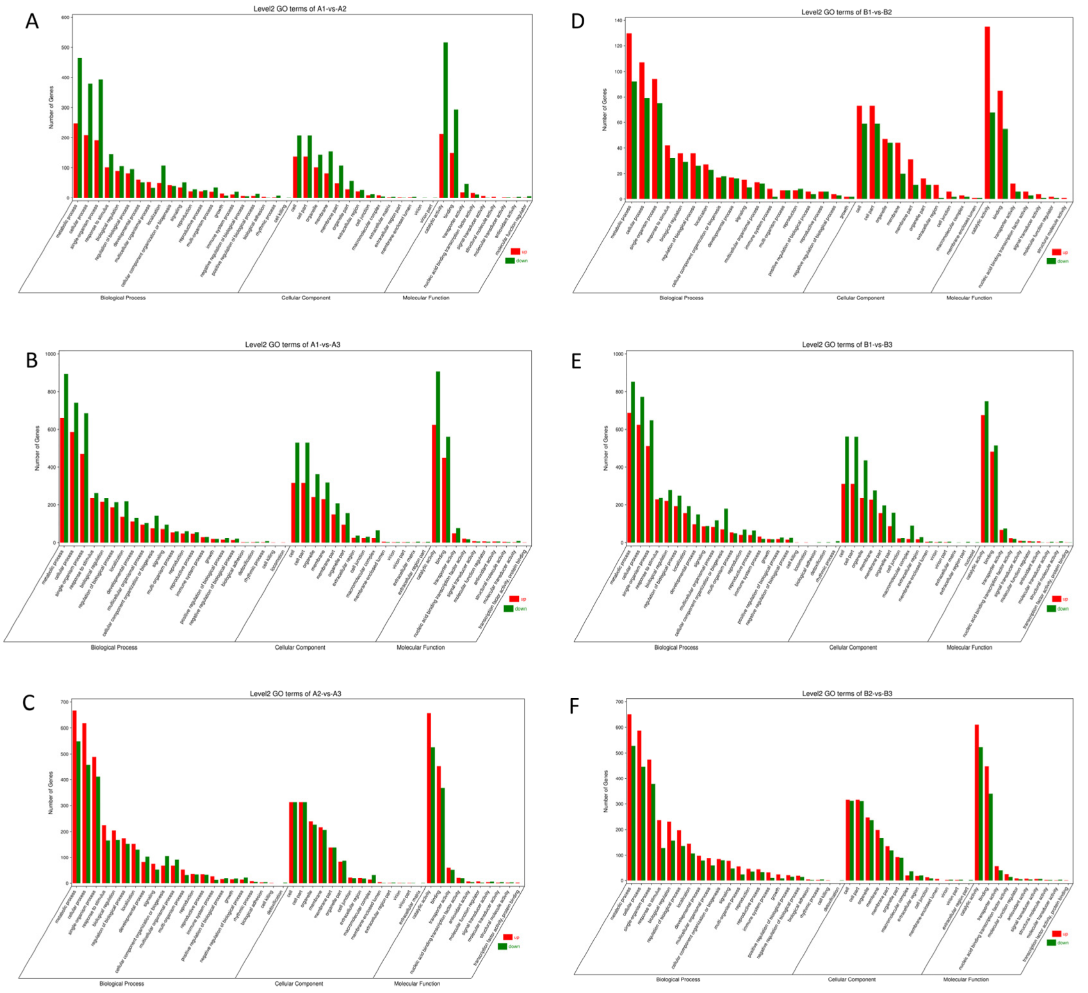

Figure S2 Gene ontology (GO) classification of DEGs in WT and M3 plants under low temperature stress conditions. The enriched biological process, cellular component and molecular function GO terms of DEGs between CK vs 2 h (A), 0 h vs 12 h (B) and 2 h vs 12 h (C) in WT plants, and DEGs between CK vs 2 h (D), 0 h vs 12 h (E) and 2 h vs 12 h (F) in WT plants.

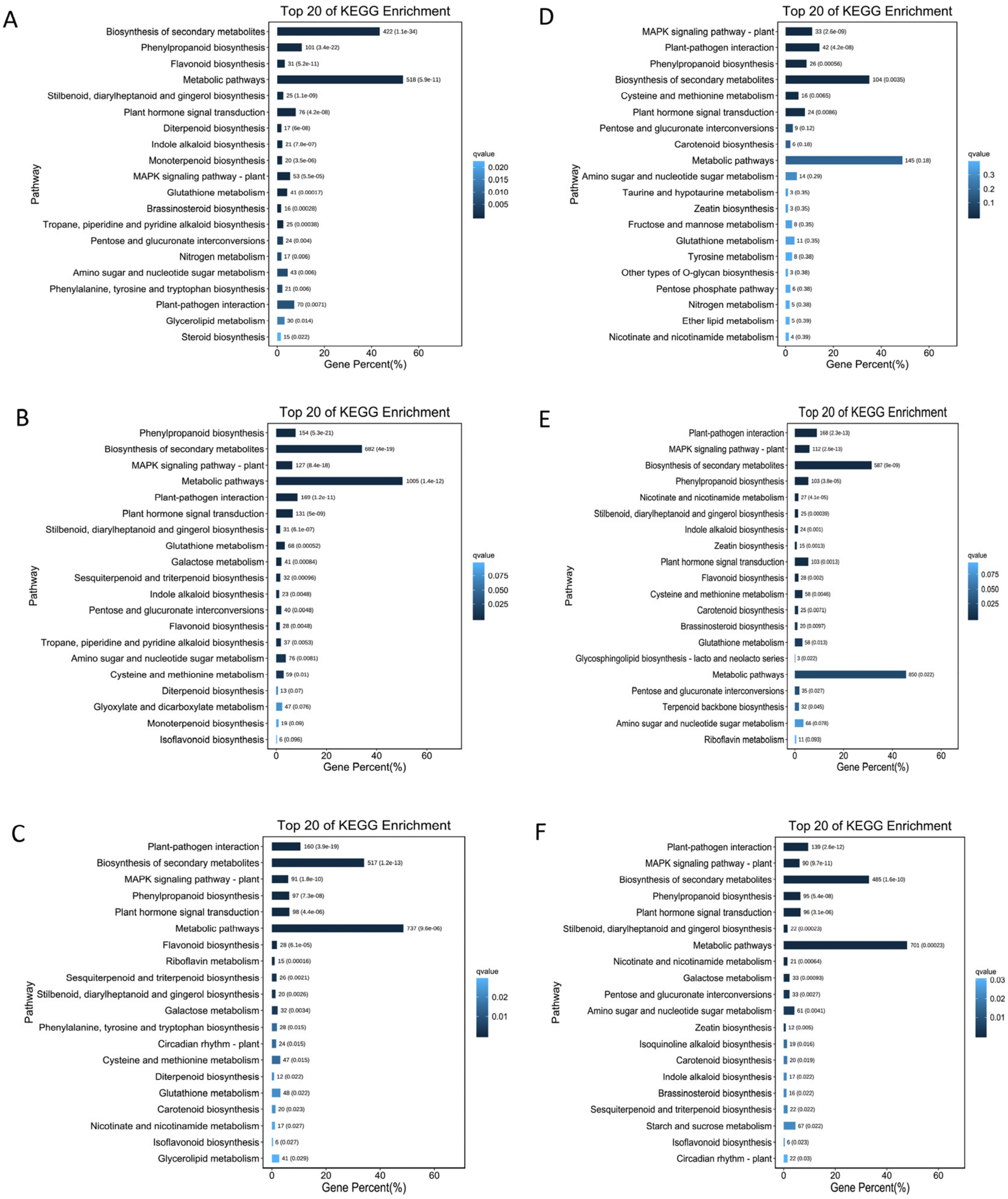

Figure S3 KEGG enrichment of DEGs in WT and M3 plants under low temperature stress conditions. The Top 20 enrichment KEGG pathway of DEGs between CK vs 2 h (A), CK vs 12 h (B) and 2 h vs 12 h (C) in WT plants, and DEGs between CK vs 2 h (D), CK vs 12 h (E) and 2 h vs 12 h (F) in WT plants.
